# Metropolitan Hospital Sunday Fund

**Published:** 1895-08-03

**Authors:** 


### Metropolitan Hospital Sunday Fund.

PARTICULARS OF THE AWARDS FOR 1895.

The following is the report of the Committee of Distribution adopted by the Council on the 29th ult.; Sir Sydney H. Waterlow in the chair :—

Your committee have the honour to recommend awards to 174 of the institutions enumerated in the appendix to this report, being an increase of sixty-nine since the first awards were made in 1873. The total amount available for distribution, after allowing for liabilities and the usual current expenses, is £44,410. Of this total £42,269 is now recommended to 121 hospitals and 53 dispensaries. Five per cent. of the total collected—viz., about £2,150—is set apart to purchase surgical appliances (in obedience to Law XII.), in monthly proportions during the ensuing year. Your committee recommend that all payments to the Fund after this date be carried to the credit of next year's Fund.

In compliance with an order of the council, and for the special use of its members, tables of statistics have been prepared as usual, showing an analysis of the number of beds in hospitals, the cost of patients both at hospitals and dispensaries, the proportionate expense of management, as well as other valuable information, and copies are sent to every member of the council.

Your committee have this year had occasion to question the admistration of no less than twenty-five institutions. Each of these have been given the opportunity of sending a deputation in conference. Your committee are, however, glad to report that in many cases the objections raised were settled by correspondence. Twelve deputations were, however, interviewed. Three institutions, not having published their accounts on the uniform system agreed upon, are ineligible. One general hospital has no award recommended, your committee not being satisfied that its council or governing body had been duly elected. Another general hospital has no award, your committee not being satisfied with its general administration. One so-called hospital, having no beds, gets no award. Another old-established hospital is found to be in no present need. One convalescent home, where the charge for maintenance of patients was deemed excessive, a small award only is recommended. One dispensary, having its accounts largely involved in mission work, is recommended only so far as your committee considered the medical work of the mission was concerned, and upon the understanding that in future the financial statements shall show the dispensary and the mission work separate. The result of other interviews will, your committee hope, lead to improvement in the future, but for the present they can only recommend awards in slightly reduced amounts.

Awards are therefore recommended to 154 institutions on full rates, to twenty-five on reduced bases. The remaining five applicants have no awards recommended.

Twenty-seven General Hospitals.

Charing Cross Hospital, £1,050; French Hospital, £410; German Hospital, £605; Great Northern Central Hospital, £375; Guy's Hospital, £700; Hampstead Hospital, £110; Italian Hospital, £80; King's College Hospital, £1,600; London Hospital, £3,750; London Homœpathic Hospital, £140; London Temperance Hospital, £700; Metropolitan Hospital, £500; Miller Hospital and Royal Kent Dispensary, £260; North-West London Hospital, £380; Poplar Hospital, £405; Queen's Jubilee Hospital, nil; Royal Free Hospital, £900; St. George's Hospital, £1,500; SS. John and Elizabeth Hospital, £100; St. Mary's Hospital, £2,000; Seamen's Hospital Society, £920; The Middlesex Hospital, £2,150; Training Hospital, Tottenham, nil; University College Hospital, £1,300; West Ham Hospital, £270; West London Hospital, £600; Westminster Hospital, £1,150.

Special Hospitals.

Five Chest Hospitals.—City of London Hospital for Diseases of the Chest, Victoria Park, £1,020; Hospital for Consumption, Brompton, £1,610; North London Consumption Hospital, Hampstead, £360; Royal Hospital for Diseases of the Chest, City Road, £400; Royal National Hospital for Consumption (Ventnor), £300.

Thirteen Children's Hospitals.—Alexandra Hospital for Hip Disease, W.C., £180; Barnet Hospital, £40; Belgrave Hospital for Children, S.W., £160; Cheyne Hospital for Incurable Children, S.W., £150; East London Hospital for Children, Shadwell, E., £500; Evelina Hospital for Sick Children, Southwark, S.E., £450; Home for Incurable Children, Maida Vale, W., £70; Home for Sick Children, Sydenham, S.E., £150; Hospital for Sick Children, Great Ormond Street, W.C., £800; North Eastern Hospital for Children, Hackney Road, N.E., £300; Paddington Green Hospital for Children, W., £150; Victoria Hospital for Children, King's Road, Chelsea, S.W., £600; "The Vine," Sevenoaks, £30.

Six Lying-in Hospitals.—British Lying-in Hospital, Endell Street, W.C., £40; City of London Lying-in Hospital, City Road, E.C., £80; Clapham Maternity Hospital, £20; East End Mother's Home, £60; General Lying-in Hospital, Lambeth, S.E., £60; Queen Charlotte's Lying-in Hospital, Marylebone Road, W., £360.

Six Hospitals for Women.—Chelsea Hospital for Women, S.W., £200; Hospital for Women, Soho Square, W., £410; Grosvenor Hospital for Women and Children, Vincent Square, S.W., £110; New Hospital for Women, Euston Road, W.C., £200; Royal Hospital for Children and Women, Lambeth, S.E., £250; Samaritan Free Hospital, Lower Seymour Street, W., £570.

Twenty-eight Other Special Hospitals.

Cancer Hospital, Brompton, S.W., £700; London Fever Hospital, Islington, N., £600; Gordon Hospital for Fistula, Vauxhall Bridge Road, S.W., £40; St. Mark's Hospital for Fistula, City Road, E.C., £60; National Hospital for the Diseases of the Heart and Paralysis, Soho Square, W., £90; Female Lock Hospital, Harrow Road, W., £180; Male Lock Hospital, Soho, W., nil; Hospital for Epilepsy, Paralysis, and other Diseases of the Nervous System, Regent's Park, N.W., £60; National Hospital for the Paralysed and Epileptic, W.C., £850; West End Hospital for Diseases of the Nervous System, £80; Central London Ophthalmic Hospital, Gray's Inn Road, W.C., £36; Royal Eye Hospital, St. George's Circus, S.E., £150; Royal London Ophthalmic Hospital, Moorfields, E.C., £560; Royal Westminster Ophthalmic Hospital, Charing Cross, W.C., £100; Western Ophthalmic Hospital, Marylebone Road, W., £33; City Orthopædic Hospital, Hatton Garden, E.C., £80; National Orthopædic Hospital, Great Portland Street, W., £48; Royal Orthopædic Hospital, Oxford Street, W., £80; Royal Sea-Bathing Infirmary, Margate, £400; Hospital for Diseases of the Skin, Stamford Street, S.E., £20; Western Skin Hospital, nil; St Peter's Hospital for Stone, Covent Garden, £50; Central London Throat and Ear Hospital, Gray's Inn Road, W.C., £55; Hospital for Diseases of the Throat, Golden Square, W.C., £30; Royal Ear Hospital, Frith Street, W., £6; Phillips Memorial Homœopathic Hospital, Bromley, Kent, £35; Dental Hospital of London, Leicester Square, W.C., £120; National Dental Hospital, 149, Great Portland Street, W., £85.

Twenty-two Convalescent Hospitals.

Metropolitan Convalescent Institution, Sackville Street, W., £650; Bexhill Convalescent Home, Sackville Street, W., £300; All Saints' Convalescent Hospital, Eastbourne, £550; Mrs. Gladstone's Convalescent Home, Woodford, £100; Hahnemann Convalescent Home, Bournemouth, £40; Hanwell Convalescent Home, £20; Herbert Convalescent Home, Bournemouth, £30; Herne Bay Baldwin Browne Convalescent Home, £25; Homœopathic Convalescent Home, Eastbourne, £10; King's College Hospital Convalescent Home, Hemel Hempstead, £90; Mrs. Kitto's Convalescent Home, Reigate, £90; London and Brighton Female Convalescent Home, £55; Mary Wardell Convalescent Home for Scarlet Fever, £101; Morley House Convalescent Home for Working Men, £210; Princess Frederica's Convalescent Home, East Molesey, £15; St. Andrew's Convalescent Hospital, Clewer, £170; St. Andrew's Convalescent Home, Folkestone, £250; St. John's Home for Convalescent and Crippled Children, Brighton, £60; St. Joseph's Convalescent Home, Bournemouth, £50; St. Leonard's-on-Sea Convalescent Home for Poor Children, £130; St. Michael's Convalescent Home, Westgate-on-Sea, £40; Seaside Convalescent Hospital, Seaford, £150.

Thirteen Cottage Hospitals.

Beckenham Cottage Hospital, £50; Blackheath and Charlton Cottage Hospital, £70; Bromley, Kent, Cottage Hospital, £75; Chislehurst, Sidcup, and Cray Valley Cottage Hospital, £40; Eltham Cottage Hospital, £30; Enfield Cottage Hospital, £40; Epsom and Ewell Cottage Hospital, £70; Hounslow Cottage Hospital, £35; Reigate and Redhill Cottage Hospital, £55; Sidcup Cottage Hospital, £30; Warley Cottage Hospital, Essex, £8; Wimbledon Cottage Hospital, £40; Woolwich and Plumstead Cottage Hospital, £30.

Five Institutions.

Establishment for Gentlewomen, Harley Street, £110; National Sanatorium for Consumption, Bournemouth, £60; Invalid Asylum, Stoke Newington, £55, Firs Home, Bournemouth, £40; St. Catherine's Home, Ventnor, £35.

Fifty-four Dispensaries.

Battersea Provident Dispensary, £70; Bloomsbury Provident Dispensary, £12; Brixton, &c., Dispensary, £60; Brompton Provident Dispensary, £27; Camberwell Provident Dispensary, £100; Camden Provident Dispensary, £14; Chelsea, Brompton, and Belgrave Dispensary, £47; Chelsea Provident Dispensary, £13; City Dispensary, £87; City of London and East London Dispensary, £20; Clapham General and Provident Dispensary, £35; Deptford Medical Mission, £15; Eastern Dispensary, £38; East Dulwich Provident Dispensary, £20; Farringdon General Dispensary, £45; Finsbury Dispensary, £55; Forest Hill Dispensary, £38; Hackney Provident Dispensary, £70; Hampstead Provident Dispensary, £43; Holloway and North Islington Dispensary, £65; Islington Dispensary, £45; Kensal Town Provident Dispensary, £9; Kensington Dispensary, £70; Kilburn, Maida Vale, and St John's Wood Dispensary, £43; Kilburn Provident Medical Institution, £30; London Dispensary, £18; London Medical Mission, Endell Street, £70; Marylebone Medical Mission, nil; Metropolitan Dispensary, £45; Notting Hill Dispensary and Maternity (Provident), £22; Paddington Provident Dispensary, £40; Pimlico Provident Dispensary (Lupus Street), £18; Portland Town Dispensary, £20; Portobello Road Provident Dispensary, £7; Public Dispensary, £50; Queen Adelaide's Dispensary, £40; Royal General Dispensary, £40; Royal Pimlico Provident Dispensary, £45; Royal South London Dispensary, £60; St. George's and St. James's Dispensary, £52; St. George's, Hanover Square, Provident Dispensary, £43; St. John's Wood and Portland Town Provident Dispensary, £30; St. Marylebone General Dispensary, £45; St. Pancras and Northern Dispensary, £45; South Lambeth, Stockwell, and North Brixton Dispensary, £45; Stamford Hill, Stoke Newington, &c., Dispensary, £55; Tower Hamlets Dispensary, £43; Walworth Provident Dispensary, £12; Wandsworth Common Provident Dispensary, £9; Westbourne Provident Dispensary and Maternity, £15; Western Dispensary, £60; Western General Dispensary, £135; Westminster General Dispensary, £31; Whitechapel Provident Dispensary, £24.

